# Bridging the Gulf: *Phytophthora* and Downy Mildews Are Connected by Rare Grass Parasites

**DOI:** 10.1371/journal.pone.0004790

**Published:** 2009-03-10

**Authors:** Marco Thines

**Affiliations:** Institute of Botany, University of Hohenheim, Stuttgart, Germany; Massachusetts General Hospital, United States of America

## Abstract

Downy mildews and root and foliar rots caused by *Phytophthora* are among the most destructive plant pathogens and therefore have attracted considerable attention during the past two decades. Although it has been realized that a close phylogenetic relationship exists, so far sharp distinction has been made between the obligate biotrophic downy mildews and the hemibiotrophic *Phytophthora*. In the study presented here, it is shown that a continuum of character states from hemibiotrophic *Phytophthora* species to obligate biotrophic downy mildews is present. Intermediate character states between downy mildews and *Phytophthora* species exist in several rare parasites of grasses, which are not embedded within the major clades of the downy mildews but are placed sister to these, with unresolved affinities to both these clades and to *Phytophthora.* They still have retained traits hitherto thought to be exclusive for *Phytophthora*. A careful review of previous research is presented and it is highlighted that uniquely for downy mildews, *Poakatesthia* may form an intracellular mycelium, growing through several host cells. In addition, scanning electron microscopy reveals that sporangiophore growth is not determinate in *Viennotia* and that outgrowth from sporangiophores is very similar to *Phytophthora infestans*. It is concluded that the sharp morphological distinction between downy mildews and *Phytophthora* species (that are often placed in separate families and even different orders), is rather artificial, since all features thought to be exclusive to *Phytophthora* or the downy mildews are united in the rare grass-parasitizing down mildew genera *Viennotia* and *Poakatesthia* and the enigmatic genus *Sclerophthora*. Therefore, several paradigms regarding the distinction between *Phytophthora* and the downy mildews need to be reconsidered.

## Introduction

*Phytophthora* species are among the most destructive rot-causing pathogens of plants, responsible for several catastrophic events, like the sudden oak death in North America, caused by *Ph. ramorum* [1] and the Irish Potato Famine caused by *Ph. infestans* [2], [3]. These pathogens can be grown on synthetic media and are hemibiotrophic in nature. The downy mildews can cause severe damage in several important crops, such grape, cucurbits, sunflower, spinach, lettuce, sorghum, millet, tobacco and spinach. Downy mildews are generally believed to be obligate biotrophic and fully dependent on living host cells – although the reasons why are still obscure – so they can not be grown on media. Downy mildews are mainly host genus or even host species specific, while *Phytophthora* species often have a much wider host range. In addition to these characteristics, there are three main morphological characteristics that are thought to distinguish *Phytophthora* species from downy mildews. First, downy mildews do not form intracellular mycelium, but invade host cells only by haustoria [4], while *Phytophthora* is able to grow through both living and dead cells [5]. Second and equally important, sporangiophore growth is terminated in downy mildews, i.e. once sporangia are formed no further growth takes place, while in *Phytophthora* the sporangiophore may grow further after sporangia have formed. Third, it is generally believed that in downy mildews all sporangia ripen simultaneously, while in *Phytophthora* sequential maturation takes place. These differences were thought to be of major importance and have been used as an argument to postulate a deep divide between downy mildews and *Phytophthora*, placing the downy mildews in a family of its own, the Peronosporaceae, while *Phytophthora* – on the basis of its thallus growth and general morphological characteristics – has usually been placed in the family Pythiaceae of the order Pythiales [6], [7]. Recent molecular phylogenetic investigations [8]–[12], however reveal that downy mildews are very closely related to a paraphyletic *Phytophthora*. Only in one study [10], was there significant support for a monophyly of the downy mildews, but this result could be attributed to an artefact due to a too narrow search radius, especially in Maximum Likelihood [13] phylogenetic reconstruction [14]. However, in all molecular phylogenies computed so far, the clade containing both downy mildews and *Phytophthora* was found to be monophyletic, often with maximum support [15], [10]. This appears at odds with the developmental and morphological differences between these two groups and the fact that *Phytophthora* infected plants usually rot and die, whilst those infected with downy mildews may remain almost or completely asymptomatic for long periods and may even recover, particularly in secondary infections of natural populations. However, the effectors secreted by these pathogens are similar [16]–[19]. The complex interactions involved in pathogenesis [20]–[22] differ mostly in respect of the final necrotic phases of infection in *Phytophthora* which is initiated by necrosis inducing proteins [23]. Although necrosis can also be observed in later stages of infection in some downy mildews, such as *Bremia*, only seldom do plants die from infections with this pathogen. *Bremia* is not culturable on artificial media and therefore it is unlikely that necrotic parts of infected plants can serve as a source of nutrition for the hyphae. In general, the downy mildews have evolved into well adapted obligate biotrophic pathogens, which may be transmitted with the seeds of their host plants and many hardly cause observable symptoms. This is especially the case in *Basidiophora* and some species of *Hyaloperonospora*, which systemically infect their hosts and in effect develop endosymbiotically [24].

Because of the close phylogenetic relationship revealed by molecular studies and the similarity in the effector genes between the hemibiotrophic *Phytophthora* and the obligate biotrophic downy mildews, the question then whether some missing links between these two groups still exist. Recent molecular phylogenetic studies [10]–[12], [25], revealed that the graminicolous downy mildews appear to be sister clades to the monophyletic major clades of the downy mildews, which contain around 95 % of all species so far described (i.e. brassicolous downy mildews – *Hyaloperonospora*, *Perofascia*; downy mildews with coloured conidia – *Peronospora*, *Pseudoperonospora*; downy mildews with pyriform haustoria – *Basidiophora*, *Benua*, *Bremia*, *Novotelnova*, *Paraperonospora*, *Plasmopara*, *Plasmoverna*, *Protobremia*). However, in none of the analyses to date did these apparent sister relationships receive high bootstrap support and therefore this molecular evidence must be treated cautiously. If the before mentioned artefact in phylogenetic reconstruction [10], [14] is taken into account, then the affinities of the graminicolous species to the other clades of the downy mildews or to *Phytophthora* remains unclear. Göker et al. [10] concluded that the evolution of the brassicolous downy mildew genera *Hyaloperonospora* and *Perofascia* had started from hosts in the Poaceae and Thines et al. [11] also noted that the possibility that downy mildew evolution in general might have started in the Poaceae cannot excluded. The basic question arising from this molecular work, addressed in this study, is whether there are any characteristics in the graminicolous downy mildews that are reflecting the close relationship of downy mildews and *Phytophthora* and the rather basal position of the graminicolous downy mildews that was revealed by recent molecular phylogenetic investigations.

## Results and Discussion

**Figure 1:**
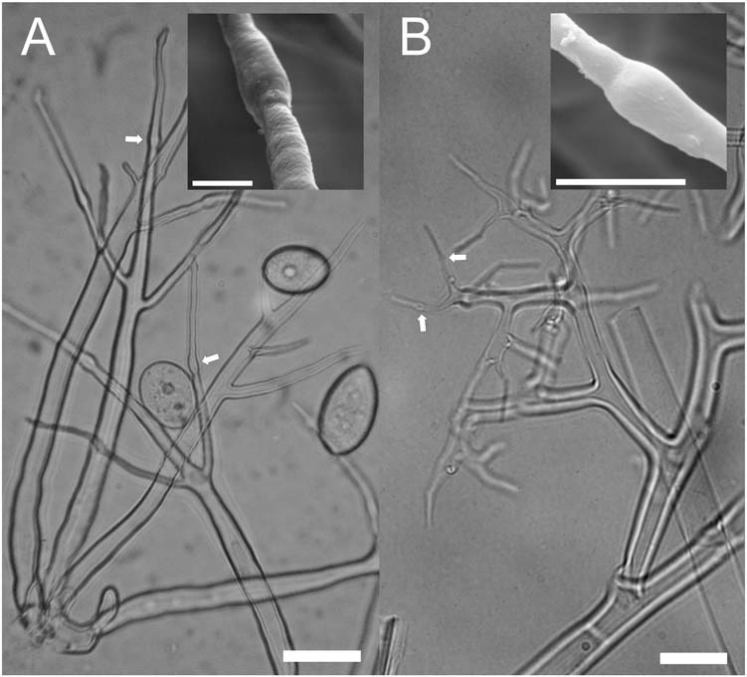
Similarity of the apophyses in *Phytophthora* and the downy mildew genus *Viennotia*. A: *Phytophthora infestans*. B: *Viennotia oplismeni*. Big pictures: Sporangiophores as seen in phase contrast light microscopy. Small pictures: Close-up of the apophyses as seen in scanning electron microscopy. Arrows point to apophyses on the ultimate branchlets, which are typical for *Phytophthora*, but in downy mildews are only present in *V. oplismeni*. Bar = 20 µm in the big pictures and 5 µm in the small pictures.

**Figure 2:**
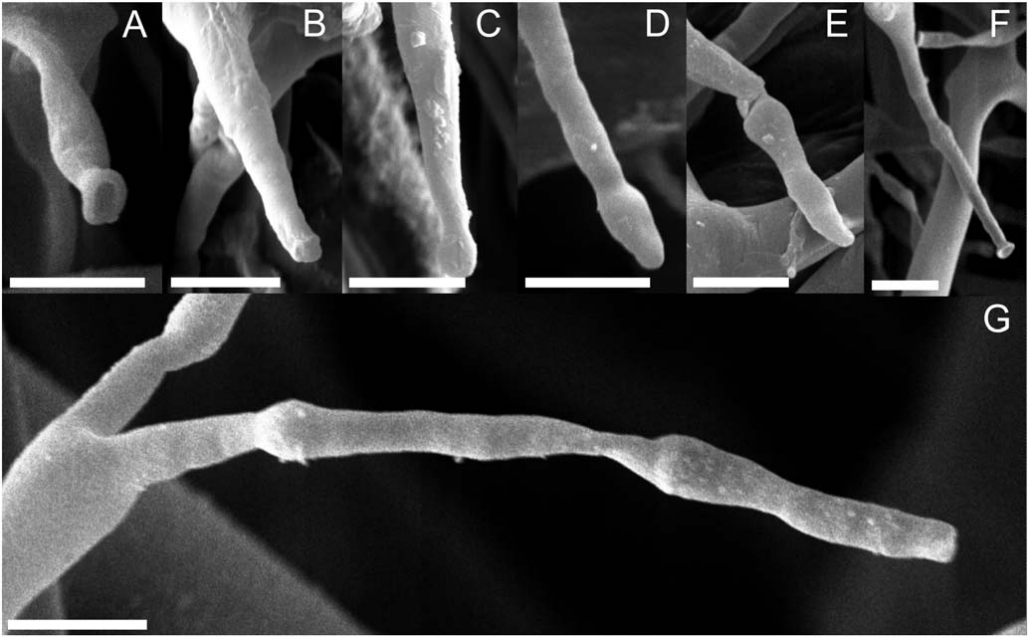
Sequence demonstrating that sporangiophore growth is not determinate in the downy mildew genus *Viennotia*. A: Primary sporangiophore tip after sporangium detachment. B–E: Sequence showing the outgrowth from the middle of the site where the sporangium had been detached. F: Secondary sporangiophore tip after sporangium detachment. G: Tertiary sporangiophore tip after sporangium detachment. Bar = 5 µm in all pictures.

In light microscopy (LM), the apophyses, which are typical for *Phytophthora*, can also be observed in *Viennotia* (Fig. 1). In scanning electron microscopy (SEM), the similarity of the apophyses in *Phytophthora* and *Viennotia* becomes even more apparent (Fig. 1). In both species, not only one, but up to three apophyses on an individual ultimate branchlet can be observed (Fig. 2). These apophyses are formed, when, after a sporangium is dispersed, the ultimate branchlet of the sporangiophore grows out again. The process of producing a sporangium, dispersal and outgrowth may repeat several times. In both *Phytophthora* and *Viennotia*, sporangiophores with three apophyses have been observed, which means that four times, a sporangium was produced on a single ultimate branchlet. The process of outgrowth and apophyse formation in *Viennotia* is illustrated in Figure 2. It should be noted that in contrast to *Phytophthora*, outgrowth takes place almost simultaneously in *Viennotia*. In both species, however, the growth of the sporangiophore is not terminated by the formation of sporangia.

The possibility for *Phytophthora*-like outgrowth in the genus *Viennotia* is unique for downy mildews. Outgrowth in *Viennotia* and *Phytophthora* takes place trough the middle of the scar left after sporangium dispersal and not as previously depicted by de Bary [26], and subsequently copied by many authors, from a swelling below the pedicel, on which the first formed sporangium may still sit [27], [28]. Scars on the apophyses that should be present if development was the result of the inflation of branchlets below the original tip could not be observed, although 500 terminal branchlets were scrutinized for each genus. Also in *Plasmopara halstedii*, abnormal sporulation with outgrowth from a sporangiophore bearing sporangia has been observed on sunflower roots by Novotelnova [29]. Determinate growth of sporangiophores in downy mildews versus indeterminate sporangiophore growth in *Phytophthora* has been considered the most important characteristic in delimitating downy mildews. Therefore the finding that in *Viennotia* sporangiophore growth is indeterminate provides important evidence that sharp distinction made between *Phytophthora* and downy mildews based on morphogenetic criteria is rather artificial.

It is generally assumed that in downy mildews hyphal growth only takes place extracellularly and only the determinate haustoria intrude into host cells, whilst in *Phytophthora* mycelium may grow through both living and dying cells. Intracellular mycelium is not uncommon in *Phytophthora* species, (e.g. *Phytophthora cinnamomi*; 5), although many species of this genus also produces digit-like haustoria. In *Poakatesthia penniseti*, a rare downy mildew parasite of *Pennisetum glaucum*, it has been observed that intracellular, callose-covered, hyphae form from haustoria, which may grow through several host cells before they enter the apoplast again [11]. Interestingly, Tippett et al. [5] have noted constrictions at sites where the intracellular mycelium passes from one cell into another which is similar to the observations made for *Poakatesthia* [11]. Intracellular mycelium apart from haustoria has so far not been reported from any other downy mildew. However, *Sclerophthora*, some basal *Pseudoperonospora* and *Peronospora* species, as well *Peronosclerospora*, which general mycelium morphology is much alike *Poakatesthia* [30], have not been investigated thoroughly in this respect. The development of an intracellular mycelium from haustoria in *Poakatesthia penniseti* raises questions regarding the obligate biotrophy of this downy mildew. Unfortunately, as only the type collection is available as a herbarium specimen, this conclusion can not be supported by cultivation experiments.

The finding of Tokura [31] that *Sclerophthora* is able to grow on artificial media is not unexpected, as there is a long-standing and ongoing debate [32]–[38], as to whether this pathogen should actually be considered a member of the genus *Phytophthora*. In a recent phylogenetic reconstructions, the placement of this genus could not be unambiguously resolved [12]; though it could be shown unequivocally that it belongs to the Peronosporales and not to the Saprolegniomycetidae, as placed by Dick [7]. That the placement of *Sclerophthora* is controversial becomes already apparent from its name: “*Sclero*” is referring to the thick-walled oospores, which are similar to those of the graminicolous downy mildew *Sclerospora* [28], [32], [34], while “*phthora*” refers to the *Phytophthora*-like vegetative mycelium including sporangiophores. The main reasons for the inclusion of *Sclerophthora* in the downy mildews have been the morphology of the oospores and the determinate sporangiophore growth. However, like in *Phytophthora*, sporangia do not form synchronously in this genus [39]. The main argument for inclusion of *Sclerophthora* in *Phytophthora* has been the similarity in the shape of sporangia und the supporting sporangiophores [35]. However, as the shape of sporangiophores and sporangia can be highly misleading characters, as has become apparent from recent molecular phylogenetic investigations in downy mildews [11], [15], [25], the exclusion of the genus from *Phytophthora* on grounds of oospore morphology might still be justified. Also the apparently fast and rather long independent evolutionary history, which can be deduced from the long branch for *Sclerophthora* in Thines et al. [12], contributes some evidence for this conclusion. Certainly, the rather isolated position of this genus on the phylogenetic trees, suggests it should not be considered to be a member of the genus *Phytophthora*.

The second downy mildew genus which has been reported to be culturable on artificial media is *Sclerospora* [40], which has always been accepted as a distinct genus. Recent phylogenetic reconstructions clearly place *Sclerospora* amongst the downy mildews [14], [15] and not with the Saprolegniomycetidae as proposed by Dick [7]. The report of cultivation by Tiwari & Arya [40] was supported by a photograph showing sporangiophores from which new sporangiophores were repeatedly arising. However, the successful cultivation of neither *Sclerophthora* nor *Sclerospora* has been reported again and so it is possible that the observations made by Tiwari & Arya [40] and Tokura [31] were fortuitous and not readily repeatable. Nevertheless, these results support the obvious assumption that obligate biotrophy has gradually evolved in the basal downy mildews until complete dependence on living host cells was established. Likewise, the synchronous development of sporangia seems to have gradually evolved, as in *Sclerophthora* the ripening of sporangia is asynchronous [39], whereas in *Viennotia*, sporangiogenesis is synchronous.

Therefore, in addition to the LM and SEM observations reported here, a careful examination of the literature also reveals that several characters states are in common for some genera of the graminicolous downy mildews with lasting sporangiophores (*Graminivora*, *Poakatesthia*, *Viennotia*), *Sclerophthora* and *Phytophthora*. Taking into account the comparison given in Table 1, it becomes apparent that not a single trait that was thought to separate the downy mildews from *Phytophthora* is always present in downy mildews and that most characteristics thought to be exclusive to *Phytophthora* can also be observed in lineages of the graminicolous downy mildews.

**Table 1:** Characteristics of downy mildews, *Phytophthora* and bridging taxa.

Characteristics of Downy mildews	Basal *Phytophthora* species	*Phytophthora infestans*	*Sclerophthora*	*Poakatesthia*	*Viennotia*	Dicot downy mildews
Sporangiophores generally emerge through stomata	−	(+)	+	+	+	+
Sporangia ripen simultaneously	−	−	−	+	+	+
Sporangiophore growth on host is determinate	−	−	+	+	−	+
Pathogens are obligate biotrophic	−	−	−	−?	+?	+
No intracellular mycelium except for haustoria	−	−	+?	−	+	+
Sporangiophores are lasting and well differentiated	−	(+)	−	+	+	+

+ = yes, − = no, () = mostly, ? not known with certainty.

### Conclusion

The three main paradigms about downy mildew distinction (obligate biotrophy, absence of intracellular mycelium, determinate sporangiophore growth) need to be rethought, as there are downy mildews species which show traits that are at variance with these basic assumptions.

The arguments listed above necessitate a critical revision of the paradigm that downy mildews are much different from *Phytophthora*. *Phytophthora* and downy mildews are both phylogenetically and morphologically connected by “bridging taxa” and share an intimate relationship. The downy mildews are therefore to be considered a more specialised sister-taxon to some advanced *Phytophthora* lineages, as they have evolved from a *Phytophthora*-like ancestor at a time, when some other basal lineages of *Phytophthora* s.l. had already parted from the downy mildew-*Phytophthora* s.str. lineage (which also includes *Phytophthora infestans*). Whether the “bridging taxa” of the graminicolous downy mildews take an intermediate phylogenetic position between *Phytophthora* and all other downy mildew clades or whether the plesiomorphic, *Phytophthora*-like, character states that can be observed in some downy mildews have been lost several times independently can so far not be resolved.

It seems likely that in the enigmatic genus *Sclerophthora* and in the rare downy mildew species *Poakatesthia penniseti* and *Viennotia oplismeni*, several pathogenicity effector genes and developmental genes intermediate to the model organisms in *Phytophthora* and the downy mildews could be found. Therefore, the investigation of these organisms might shed light on the shift from hemibiotrophy to obligate biotrophy and thereby help to decipher the most basal chapter in downy mildew evolution.

## Materials and Methods

### Oomycete material

Of the extremely rare parasites Poakatesthia penniseti and Viennotia oplismeni only the type collections are available (IMI 137328c and IMI 103944, respectively), which were investigated in cause of this study. The Phytophthora specimen depicted in this study is deposited in the herbarium of the University of Hohenheim (HOH) as HUH 992.

### Microscopy

For light microscopy (LM) of sporangia and sporangiophores, small pieces of the samples were transferred to 5% chloral hydrate to restore turgidity. Preparation for scanning electron microscopy was done as previously described [41].
